# Supplementary material for: Plasmons in the Kagome metal CsV3Sb5
Source: Nat Commun. 2024 Jun 25;15:5389. doi: 10.1038/s41467-024-49723-x (PMC11199534; doi:10.1038/s41467-024-49723-x)
Supplement: Supplementary file 1 — Supplementary Information [file 41467_2024_49723_MOESM1_ESM.pdf]

Supplementary Information

Plasmons in the Kagome Metal CsV_3Sb_5

H. Shiravi[1,2], A. Gupta[1,2], B. R. Ortiz[3,4], S. Cui[1,2], B. Yu[5], E. Uykur[6,7], A. A. Tsirlin[8], S. D. Wilson[3], Z. Sun[5,†], G. X. Ni[1,2,†]

[1]Department of Physics, Florida State University, Tallahassee, Florida 32306, USA.
[2]National High Magnetic Field Laboratory, Tallahassee, Florida 32310, USA.
[3]Materials Department, University of California Santa Barbara, Santa Barbara, California, 93106, USA.
[4]Materials Science and Technology Division, Oak Ridge National Laboratory, Oak Ridge, 37831, Tennessee, USA.
[5]State Key Laboratory of Low-Dimensional Quantum Physics and Department of Physics, Tsinghua University, Beijing 100084, China.
[6]Physikalisches Institut, Universit¨at Stuttgart, 70569 Stuttgart, Germany.
[7]Helmholtz-Zentrum Dresden-Rossendorf, Institute of Ion Beam Physics and Materials Research, 01328 Dresden, Germany.
[8]Felix Bloch Institute for Solid-State Physics, Leipzig University, 04103 Leipzig, Germany
[†]Corresponding Author: zysun@tsinghua.edu.cn, guangxin.ni@magnet.fsu.edu.

Supplementary Note List:

1. **Device fabrication and nano-IR characterization**
2. **Origin of the plasmonic fringes**
3. **Extracting the plasmonic wavelength**
4. **Hyperbolic plasmons vs. normal surface plasmons**
   a. **The dispersion of plasmons in a slab**
   b. **Surface plasmons ($\epsilon_z < 0$)**
   c. **Hyperbolic plasmons ($\epsilon_z > 0$)**
5. **Electronic band structure and optical conductivity from DFT**

Supplementary Note 1: Device fabrication and nano-IR characterization

All of our CsV_3Sb_5 microcrystals were obtained using mechanical exfoliation. The typical atomic force microscope (AFM) topography image of thin CsV_3Sb_5 flakes is shown in Supplementary Figure 1a (SFig. 1a). SFig. 1b shows the corresponding near-field image at the selected IR frequency. Clear plasmon polariton waves occupy the whole field of view, which are discussed in the main text and also below. For CsV_3Sb_5 hyper-lensing studies, a substrate with prepatterned Au microstructures defined via standard E-beam lithography was utilized during the exfoliation steps (main text, Fig. 4).

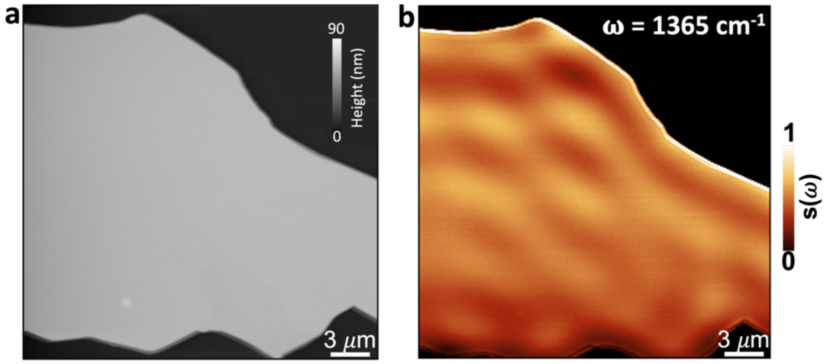

Supplementary Figure 1 | AFM topography and $s(\omega)$ image of a CsV_3Sb_5 flake. (a) AFM topography imaging of the CsV_3Sb_5 sample under study. (b) $s(\omega)$ image of the CsV_3Sb_5 flake at the selected IR frequency of 1365 cm^{-1}.

Besides natural crystal edges, prepatterned gold antennas underneath the crystal can also effectively launch hyperbolic polaritons [S1]. As a result, hyper-lensing imaged structures are expected to emerge on the top surface of the sample flakes. In the main text Fig. 4, we show the topography and near-field images of a CsV_3Sb_5 crystal covering the micro-sized gold pattern. The hyperbolic wave pocket develops along the Au antenna physical boundaries over a wide range of frequencies, consistent with natural edge launched polariton results as discussed in the main text. Moreover, as one of the hallmarks of hyper-lensing effect, the underlying structure appears with a varying outline that is frequency-dependent due to its hyperbolic dispersion. This is exactly our frequency-dependent results shown. Specifically, we observed a systematic expansion of the underlying structure as the incident light wavelength varies from 6.25 μm (1600 cm^{-1}) to 10 μm (1000 cm^{-1}), as shown in Fig. 4.

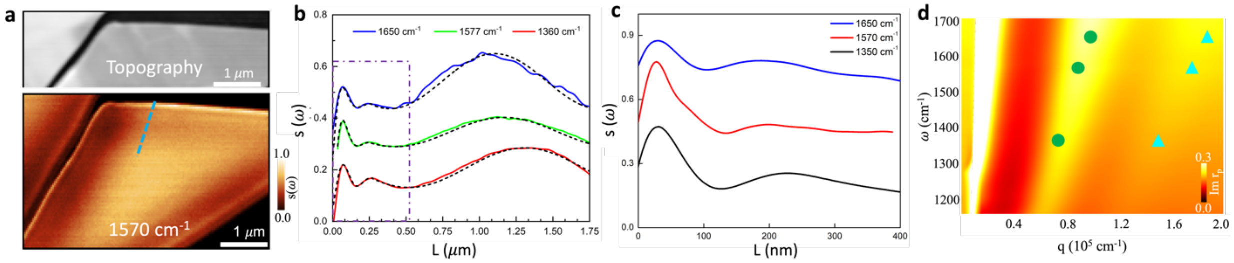

Supplementary Figure 2 | nano-IR imaging and $s(\omega)$ line profiles of a CsV_3Sb_5 flake. (a) AFM topography and $s(\omega)$ image of a thin CsV_3Sb_5, with the blue dashed line representing the line-cuts across the images. (b) The $s(\omega)$ line profiles of the CsV_3Sb_5 flake at different IR frequencies, with dashed box indicating the enlarged regime shown in panel c. (c) A Zoom-in version of the line profiles to highlight the higher-order resonances. (d) $\mathrm{Im}[r_p]$ for a 17 nm flake with $\epsilon_c = 0.35$. The extracted experimental data are displayed as green dots for the first-branch

and cyan triangles for the higher-order mode, respectively.

The obtained higher-order hyperbolic plasmon polariton modes are readily comparable with the calculated dispersion in the frequency-momentum (ω, q_p) space in SFig. 2d. In SFig. 2, we show the AFM topography and the associated near-field amplitude s(ω) images of a thin CsV_3Sb_5 flake (17 nm) obtained at different IR frequencies. In addition to the principal plasmon mode, we observe another plasmon fringe pattern appears near the sample edge. This can be directly visualized in both nano-IR imaging and the extracted plasmonic line-profiles across a wide range of frequencies (SFig. 2). We also wish to note that one key distinction between the observed higher-order mode and the edge-mode is the noticeable difference in their plasmonic wavelengths. In the higher-order mode, the corresponding plasmon wavelength is one order of magnitude shorter than the principle hyperbolic mode.

Supplementary Note 2: Origin of the plasmonic fringes

The λ_p fringes come from two possible paths. In the first path, the AFM tip acts as a launcher for plasmonic waves of wavelength λ_p that propagate radially outwards from the SNOM tip. After arriving at the sample edge, these plasmons are partially converted into far-field photons and then collected by the detector. The second path is the reverse of the first [S2], see SFig. 6.

In addition to these two paths, there is another possible contribution which leads to $\lambda_p/2$ fringes [S3]. In this contribution, the far-field photons are converted into plasmon polaritons at the location of the tip, which propagate to the sample edge before being reflected back to the tip. The reflected plasmons are then scattered to the detector by the tip. In previous studies of graphene plasmons, this path has been extensively discussed [S4-S6]. However, in the current work, we believe this photon path is unlikely to play the dominant role. If the observed fringes were indeed $\lambda_p/2$ fringes, the near-field period $l = \lambda_p/2$ would be shorter than half of the vacuum photon wavelength $\lambda_{IR}/2$. This contradicts our experiment since the observed fringe period is systematically large than $\lambda_{IR}/2$.

Moreover, we performed nano-IR imaging studies on samples with prepatterned metallic micron-sized structures on top of the CsV_3Sb_5 flakes. These prepatterned structures located in the center of the flake serve as fixed plasmonic antennas that predominantly produce fringes with λ_p periodicity. In SFig. 3, we show the experimental results observed from the prepatterned micron-

size structures. By extracting the real-space plasmon line-profiles as depicted in SFig. 3, we found the corresponding plasmon wavelength matches well with the edge-associated plasmon wavelength. This leads us to conclude that the plasmon waves near the physical edges have λ_p periodicity.

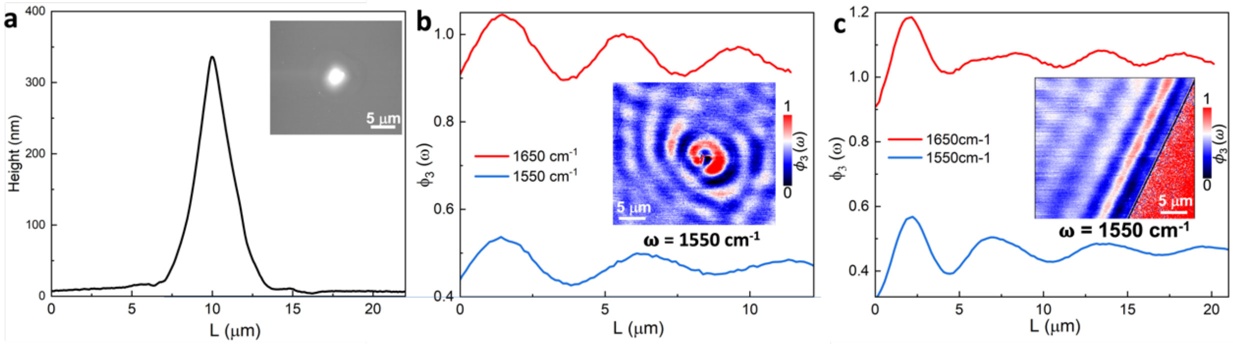

SFigure 3. Nano-IR image studies of pre-patterned metallic structure on top of a 400 nm thick CsV_3Sb_5 flake. Panel a shows the line-profile of the structure's height and morphology, with diameter of 2 μm, alongside an inset showing the AFM topography image. Panel b and c show corresponding nano-IR images, obtained in the vicinity of the pre-patterned disk and the flake edge at different IR frequencies. By comparing the wavelength obtained in panels b and c, we can firmly establish that the plasmon fringes near the edge have wavelength periodicity of λ_p.

In the following, we also briefly discuss another possible alternative explanation for the observed fringe pattern, which could be the formation of a plasmonic resonator. A plasmonic resonator is a structure that can support localized surface plasmon resonances through the collective oscillation of free electrons in response to light. In general, there are two ways to realize a plasmonic resonator in thin flakes of metal: i) one way is to create a patterned structure on its surface, such as an array of nanostructures or a periodic array of holes, which can confine light and enhance the local electric field. The size, shape and spacing of these structures can be engineered to tune to the specific resonance frequency and intensity. ii) another way is to introduce defects or impurities which can create local regions of different refractive index and hence modify the plasmonic propagation and scattering. For instance, a single defect or vacancy in a monolayer graphene can act as a quantum dot and induce plasmon resonances in the surrounding metal/semimetal layers.

However, in our samples, there are neither patterned arrays of nanostructures nor pre-defined single defect or vacancy as local resonance centers. It is also challenging to realize a resonator based on a pristine thin flake that can maintain its resonances over a very wide range of frequencies,

from 6-11 μm and beyond. Therefore, we think it is unlikely that the resonator can naturally forming in CsV_3Sb_5 flakes without any extra fabrication processes. Instead, the interpretation of the data within the edge-reflected/launched plasmon scenario fits well with our experimental observations, which provides a reasonable explanation for our results.

Supplementary Note 3: Extracting the plasmonic wavelength

To precisely determine the plasmon wavelength λ_p, we have performed Fourier transform (FT) as follows. We extracted the average line-profiles perpendicular to the fringes, and then performed FT analysis on these fringe profiles. The resulting plasmon fringe line-profiles and the corresponding FT profiles are shown in SFig. 4. Since the observed fringe period is clearly larger than 1 μm, we attribute the FT peak below 1 μm^{-1} to λ_p. These propagating waves with a plasmonic wavelength of λ_p are collected by the SNOM tip, which serves as the polaritonic wave receiver [S2].

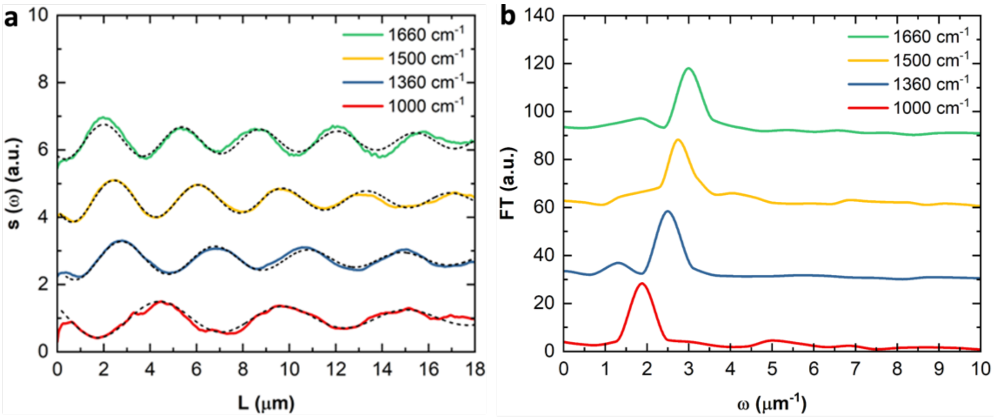

Supplementary Figure 4 | $s(\omega)$ Line-profiles and Fourier transformation (FT) (a) s(ω) line profiles of a 380 nm thick CsV_3Sb_5 flake at various IR- frequencies. (b) FT of the corresponding line profiles.

It is worth noting that the obtained plasmon wavelengths for vertical and horizontal sample configurations are slightly different from each other, especially for thicker crystals. In SFig. 5, we show the nano-IR imaging results obtained from a thicker flake with a thickness of 380 nm. The wavelength obtained from the vertical configuration is slightly shorter than the wavelength obtained from the horizontal configurations, as indicated in both the plasmon line-profiles and the FT results (SFig. 5).

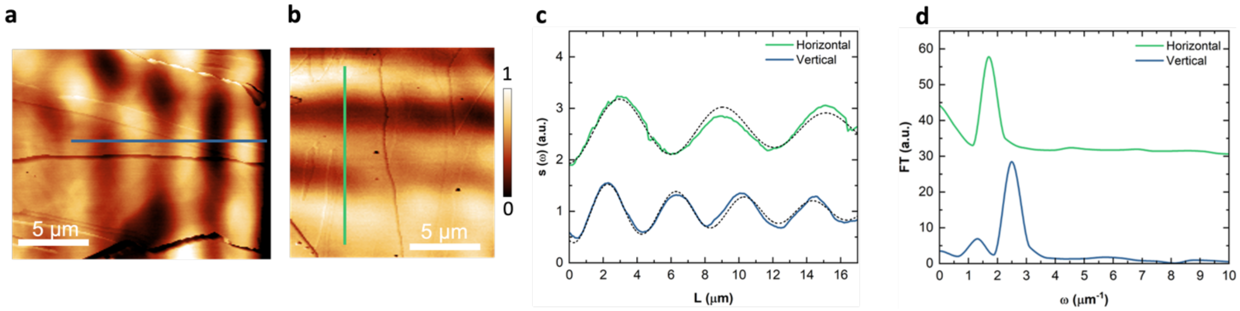

Supplementary Figure 5 | Nano-IR image and $s(\omega)$ line profile of a CsV_3Sb_5 flake with different orientations. Nano-IR image of a 380 nm thick CsV_3Sb_5 flake, with (a) a vertically oriented and (b) a horizontally oriented flake. The blue and green solid lines indicates the locations of the line-cuts. (c) The corresponding s(ω) line profiles from a&b are shown in (c), and the FT of the corresponding line profiles is shown in (d).

The reason is that there are two paths contributing to the signal: one path involves photons scattered directly back to the detector by the tip; the other path involves photons converted into propagating plasmons by the tip and then scattered back to the detector after reaching the edge of the sample (and the other way around). The observed signal is the interference between the electric fields from these two paths. Therefore, the fringe patterns with a period $\lambda^* = 2\pi/k^*$ has contributions not only from the plasmons (the matter component), but also from the far-field photons (the light component). Here,

$$\boldsymbol{k}^* = \boldsymbol{q} + \boldsymbol{q}_0 \rightarrow k^* = q + k_0 \sin\alpha \cos(\phi - \phi_{\text{plasmon}}), \quad (1)$$

whereas q_p is the momentum of the observed polaritonic modes, k_0 is free-space photon wavevector, $\alpha = 60^{\circ}$ is the incident angle of the incoming beam, and $\phi = 74.8^{\circ}$ is the azimuthal angle of the incident beam relative to the x direction (SFig. 6). In the current experiment, the second term stemmed from the light component cannot be neglected. In fact, it is the variation of the azimuthal angle ϕ that causes the variation of the obtained wavelengths. During the data analysis, the second term as the far-field factor was subtracted in order to obtain the pure plasmon momentum/wavelength information. Accordingly, the plasmon wavelength λ_p was extracted using the following equation:

$$\lambda_p = \frac{1}{\frac{1}{\lambda^*} - \frac{1}{\lambda_0}\sin\alpha\cos(\phi - \phi_{\text{plasmon}})} \quad (2)$$

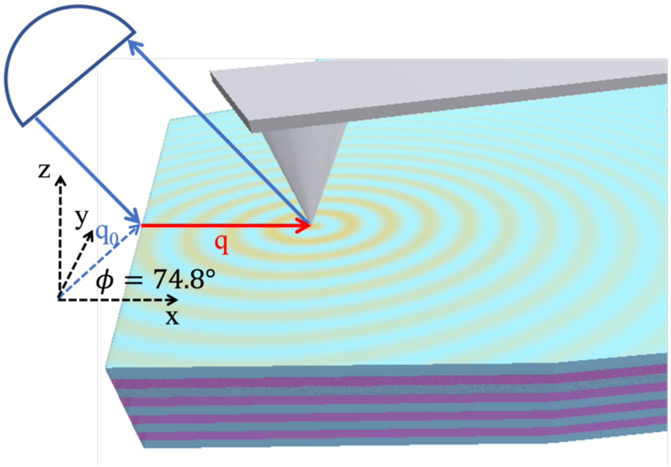

Supplementary Figure 6 | The schematic of the near-field setup. Illustration of how the real-space fringes can have contributions from both the plasmon momentum and that of far-field photons.

Supplementary Note 4: Hyperbolic plasmons vs. normal surface plasmons

Being a metallic compound, it is natural to expect that propagating surface plasmons could also emerge on the surface of CsV_3Sb_5. To this end, we have carried out a systematic analysis with the goal of searching for conventional surface plasmons and comparing them with our experimental results. In the following, we present the calculated dispersions of normal surface plasmons and hyperbolic plasmons for detailed comparison.

a. The dispersion of plasmons in a slab

For simplicity, we show the analytical results for a uniaxial slab embedded in vacuum. The condition for the plasmon modes of the slab is (see Appendix B of Ref. [S16], Eq. 1 of Ref. [S3] and Sec. 4 of its supplemental material):

$$r_p^2 e^{i2k_z d} = 1, \qquad r_p = \frac{\frac{k_z}{\epsilon_\perp} - k_{sz}}{\frac{k_z}{\epsilon_\perp} + k_{sz}} \tag{3}$$

where $k_z = \sqrt{\epsilon_\perp k_0^2 - \frac{\epsilon_\perp}{\epsilon_z} q^2}$ is the z-direction wave vector of the electromagnetic wave inside the slab, $k_{sz} = \sqrt{\epsilon_s k_0^2 - q^2}$ is the z-direction wave vector of the wave in vacuum, $\epsilon_\perp / \epsilon_z$ is the in-plane/out-of-plane dielectric, d is the thickness of the slab, $k_0 = \omega / c$ is the wavelength of vacuum photons at frequency ω, and q is the in-plane momentum of the plasmons. r_p is the reflection coefficient of the wave inside the slab upon the top and bottom surfaces. The above equation is

equivalent to the condition of poles in the reflection coefficient of the whole slab to external fields and has a simple interpretation: the wave must return to itself after a round trip inside the slab.

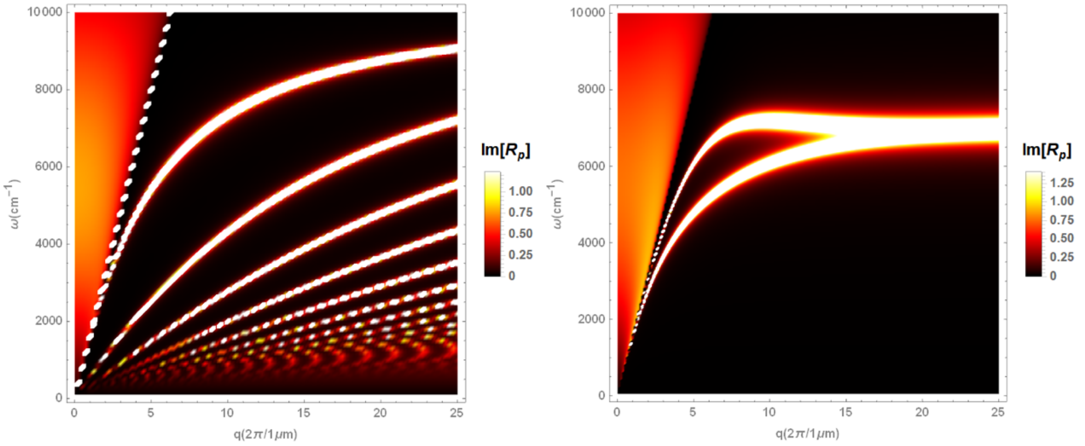

Supplementary Figure 7 | Left: The imaginary part of the near-field reflection coefficient $\mathrm{Im}[r_p]$ of a metallic slab with thickness of 200 nm. The bright dispersion peaks are the hyperbolic plasmonic modes. In this plot, the in-plane dielectric function is $\epsilon_{ab} = 1 - \frac{\omega_p^2}{\omega(\omega+i\gamma)}$ with $\omega_p = 10^4\ \mathrm{cm}^{-1}$ while the out-of-plane dielectric is $\epsilon_c = 1$. Right: Same as left except that $\epsilon_c = -1$. In this case, only two dispersion peaks appear corresponding to the surface plasmons as detailed in the text.

b. Surface plasmons ($\epsilon_z < 0$)

If the slab were not hyperbolic, meaning $\epsilon_x, \epsilon_z < 0$, there are two branches of plasmons:

$$q_\pm^2 = \frac{1 - \frac{1}{\epsilon_x}\delta_\pm}{1 - \frac{1}{\epsilon_x \epsilon_z}\delta_\pm} k_0^2 \tag{4}$$

where $\delta_+ = \frac{1+e^{ik_z d}}{1-e^{ik_z d}}$ corresponds to the symmetric branch, $\delta_- = \frac{1-e^{ik_z d}}{1+e^{ik_z d}}$ corresponds to the anti-symmetric branch. We note that $ik_z d$ has a real negative component. The right panel of SFig. 7 shows a typical example of these two branches in a slab made of a simple anisotropic Drude metal. The fact that the thickness d affects the dispersion has a simple interpretation: the plasmons from the top and bottom surfaces couple to each other with a coupling strength dependent on d, and hybridize into the two branches.

We first discuss the modes close to the light cone. Assuming $|ik_z d| \gg 1$ (which is easily met considering $k_z = \sqrt{\epsilon_\perp k_0^2 - \frac{\epsilon_\perp}{\epsilon_z} q^2}$ and that $\epsilon_\perp$ is a large negative number), one has $|e^{ik_z d}| \ll 1$

and $\delta_\pm \approx 1$ such that one may neglect its q dependence, simplifying the dispersion to

$$q_\pm^2 = \frac{1-\frac{1}{\epsilon_x}}{1-\frac{1}{\epsilon_x \epsilon_z}} k_0^2 \xrightarrow{|\epsilon_x| \gg 1} \left(1 - \frac{1}{\epsilon_x} + \frac{1}{\epsilon_x \epsilon_z}\right) k_0^2. \tag{5}$$

That means both branches are very close to the light cone. Experimentally, $\epsilon_x \sim -200$ in the relevant frequency range while the measured plasmon momentum are apparently away from the light cone, thus ruling out the possibility of surface plasmons.

For the plasmons far away from the light cone ($q \gg k_0$), $\delta_\pm$ will be super close to 1. Therefore, to satisfy $q \gg k_0$, one requires $\epsilon_x \epsilon_z \to 1$, which means the two branches of plasmons approach the same constant frequency fixed by $\epsilon_x \epsilon_z \approx 1$, as shown in the right panel of SFig. 7. In the isotropic case, this means $\epsilon_x(\omega) \approx -1$, recovering the familiar condition for surface plasmons.

To be more concrete, we have performed $r_p = r_p(\omega, q)$ calculations and constructed the frequency-momentum (ω, q_p) dispersion of CsV_3Sb_5, as shown in SFig. 8. Specifically, the $\mathrm{Im}[r_p]$ shown in SFig. 8 is calculated for CsV_3Sb_5 residing on SiO_2/Si substrate using the experimentally obtained in-plane dielectric function $\epsilon_{\mathrm{ab}}(\omega)$ (~ -200 in the relevant frequency range) [S7] and the out-of-plane dielectric function $\epsilon_{\mathrm{c}}(\omega)$ from DFT calculations. In order to search for these two branches of surface plasmons, a variable of sample thickness has been considered. It is found that only when the CsV_3Sb_5 is thin enough (< 20 nm), these two surface plasmon branches can appear close to the light cone. For the thickness of 50 nm (SFig. 8d), these two modes have been damped out. The results presented in SFig. 8 provides further evidence that the experimentally observed modes are not typical surface plasmons.

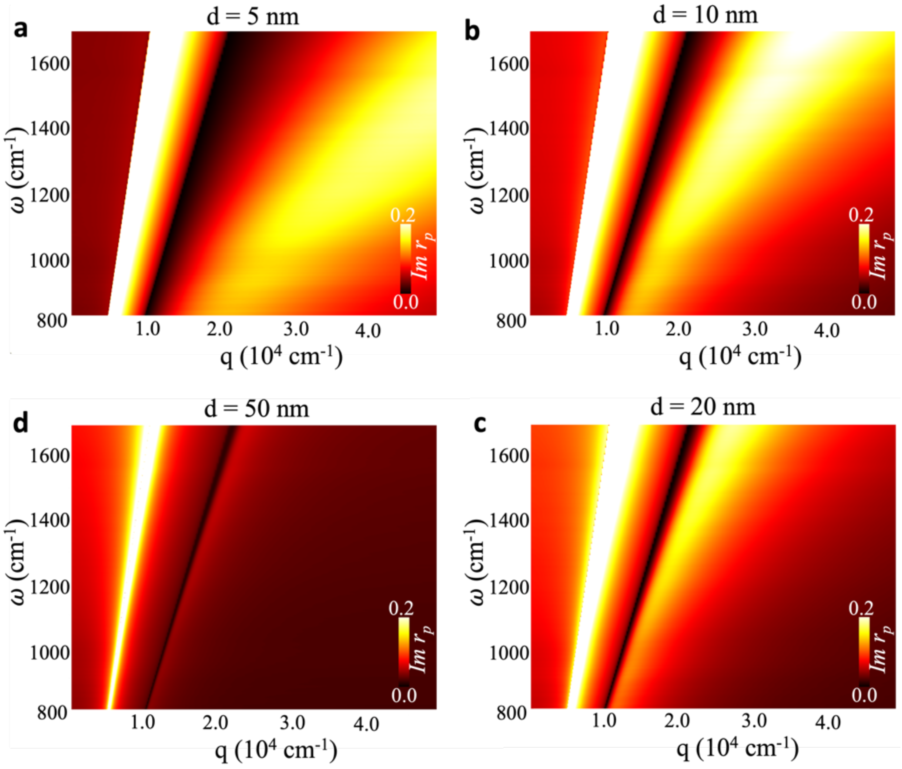

Supplementary Figure 8 | The frequency-momenta dispersion (r_p) of the two surface plasmon branches for a CsV_3Sb_5 slab with various thicknesses shown in panel a-d.

c. Hyperbolic plasmons ($\epsilon_z > 0$)

In this case, k_z in Eq. (3) is real. The dispersion condition (Eq. (3)) for the modes can be written as

$$2k_z d + 2\delta = 2N\pi \quad (6)$$

where $e^{i\delta} = r_p$ and $\delta(\omega, q) = 2\text{ArcTan}[\frac{\mathrm{i}k_{sz}}{k_z/\epsilon_\perp}]$ means the reflection phase shift of the wave upon the top and bottom surfaces. This above condition has a simple interpretation: the accumulated phase of a round trip has to be integer times 2π in order to form an eigenmode.

We may proceed by making certain approximations for the phase shift. Since $\epsilon_\perp$ is a large negative number in the frequency range of interest, one has $\frac{\mathrm{i}k_{sz}}{k_z/\epsilon_\perp} \gg 1$ such that $\delta(\omega, q) \approx \pi - 2\frac{k_z/\epsilon_\perp}{\mathrm{i}k_{sz}}$. For the principle (zeroth) mode, $2k_z d$ just needs to compensate the small $-2\frac{k_z/\epsilon_\perp}{\mathrm{i}k_{sz}}$ term to achieve a net phase shift of 0. For higher order modes, the small $-2\frac{k_z/\epsilon_\perp}{\mathrm{i}k_{sz}}$ term can be neglected. Plugging

in the expression $k_z = \sqrt{\epsilon_\perp k_0^2 - \frac{\epsilon_\perp}{\epsilon_z} q^2}$, the dispersion of the zeroth and Nth branches may be approximated by

$$q_0^2 = \frac{4}{\epsilon_\perp^2}\frac{1}{d^2} + k_0^2, \qquad q_N^2 = -\frac{\epsilon_z}{\epsilon_\perp}\left(\frac{N\pi}{d}\right)^2 + \epsilon_z k_0^2. \tag{7}$$

The zeroth branch is very close to the light cone as long as $|\epsilon_\perp| d \gg \lambda_0$ where $\lambda_0 = 2\pi/k_0$ is the wavelength of vacuum photons. Therefore, the observed plasmons are most likely the $N = 1$ branch hyperbolic plasmons. For thin slabs, $d \ll \frac{\lambda_0}{\sqrt{4|\epsilon_\perp|}} \sim 300$ nm, the first term in the $N = 1$ dispersion dominates, such that the imaginary part of q_1 is determined by the highly dissipative $\epsilon_\perp$, rendering the plasmons strongly damped. For thick slabs, $d \gg \frac{\lambda_0}{\sqrt{4|\epsilon_\perp|}} \sim 300$ nm, the second term dominates, such that the plasmon damping is determined by the imaginary part of ϵ_z only, which could be quite small. This is consistent with the experimental observation. A physical explanation for the low loss of the plasmons in the thick slab is shown in SFig. 9. The observed $N = 1$ plasmons have an effective z-direction momentum of $k_z \approx \frac{\pi}{d}$, meaning they lie quite close to the dome of the iso-frequency surface. In this part of the dispersion, the electric field of the mode is mainly along z-direction, such that the damping is determined by ϵ_z only, immune to the high in-plane loss.

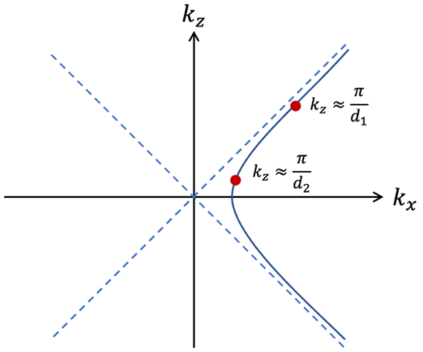

Supplementary Figure 9 | The schematic momenta of hyperbolic plasmons (first branch) on the iso-frequency surface for a thick slab with thickness d_2 and a thin one with thickness d_1. For the thick slab, the plasmon momentum is mainly in-plane, so that the electric field is almost along z-direction, meaning its damping rate is controlled by ϵ_z, immune to the high loss of in-plane response.

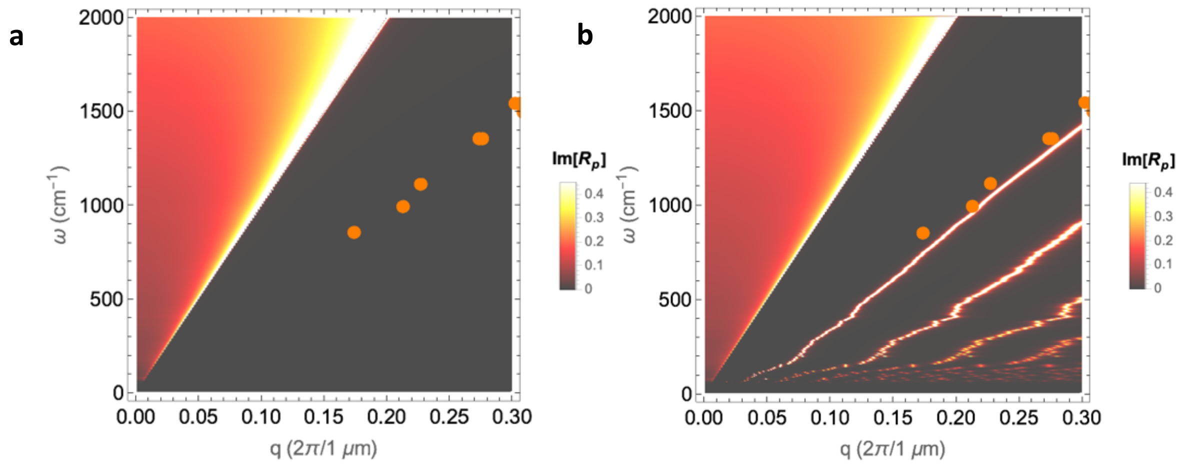

Supplementary Figure 10 | (a) r_p of a 380 nm thick slab for $\epsilon_c = -3.0$ and ϵ_{ab} being that of CsV_3Sb_5 but with greatly reduced imaginary part. (b) Same as (a) but with $\epsilon_z = 3.0$. The dots are experimental dispersion on a slab of the same thickness.

If the observed plasmons are the $N = 1$ branch hyperbolic plasmons, given $\epsilon_\perp$ measured from far-field optics and the complex q measured from scanning near-field optics, one can invert the dispersion for the first branch to obtain ϵ_z:

$$\epsilon_z = \frac{q_1^2}{-\frac{1}{\epsilon_\perp}\left(\frac{\pi}{d}\right)^2 + k_0^2}. \qquad (8)$$

The extracted $\epsilon_z(\omega)$ is plotted in Fig. 5 of the main text.

In the case of the simplest in-plane Drude metal ($\epsilon_z = 1, \epsilon_\perp = 1 - \frac{\omega_p^2}{\omega^2}$), for $q \gg k_0$ and $\omega \ll \omega_p$, the dispersion simplifies to

$$qd = 2\frac{\omega^2}{\omega_p^2} + N\pi\frac{\omega}{\omega_p} \qquad (9)$$

It is obvious that the zeroth branch is like a plasmon ($\omega \sim \sqrt{q}$) while the higher order branches have linear dispersions ($\omega \sim q$), as shown in the left panel of SFig. 7.

Supplementary Note 5: Electronic band structure and optical conductivity from DFT

Real and imaginary parts of $\epsilon_z(\omega)$ were obtained from DFT calculations performed for the experimental crystal structure of CsV_3Sb_5. Self-consistent calculations in Wien2K [S8] with the Perdew-Burke-Ernzerhof exchange-correlation potential [S9] were converged on the k-mesh with 36×36×18 points in the first Brillouin zone. Consequently, dielectric function and optical

conductivity were calculated using the optic routine of Wien2K [S10] on the dense k-mesh with 80×80×40 points. This calculation yields the inter-band contribution to the optical conductivity. SFigure 10 shows the calculated band structure of CsV_3Sb_5, which is consistent with the earlier report [S7] and reveals band saddle points near the M-point at 50-100 meV below the Fermi level. The out-of-plane optical conductivity (σ_{zz}) with fewer absorption peaks at low energies is much lower than the in-plane optical conductivity (σ_{xx}). In general, the characteristic feature of σ_{xx} is the appearance of three absorption peaks at 30-140 meV due to inter-band transitions in the vicinity of the M-point. Only the lower of these three transitions leads to a distinct peak in σ_{zz}. Likewise, the absorption feature around 700 meV (optical transitions to the Kagome flat bands lying at 600-800 meV) is hardly traceable in σ_{zz}.

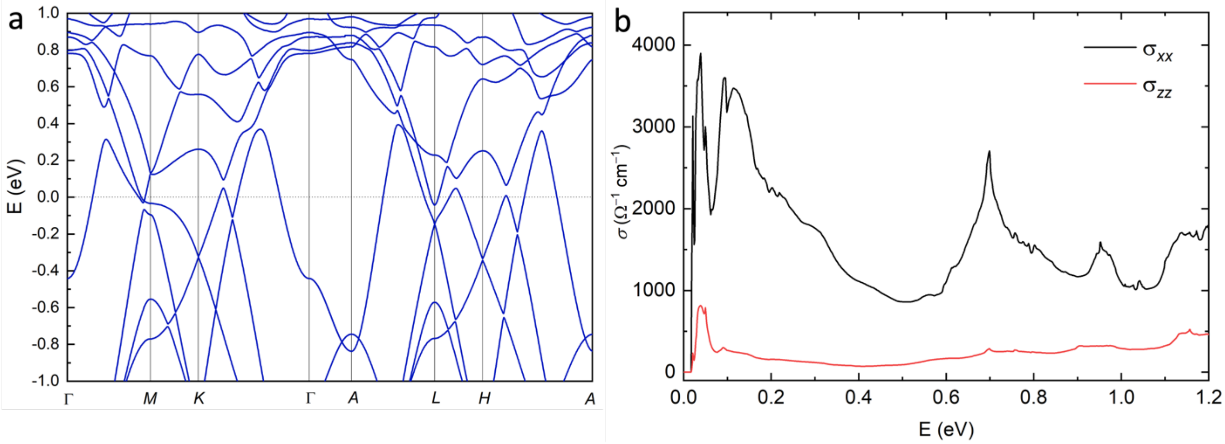

Supplementary Figure 11 | (a) DFT calculated band structure of CsV_3Sb_5, with the Fermi level set at 0 eV. (b) DFT calculated real parts of the in-plane (σ_{xx}) and out-of-plane (σ_{zz}) optical conductivity.

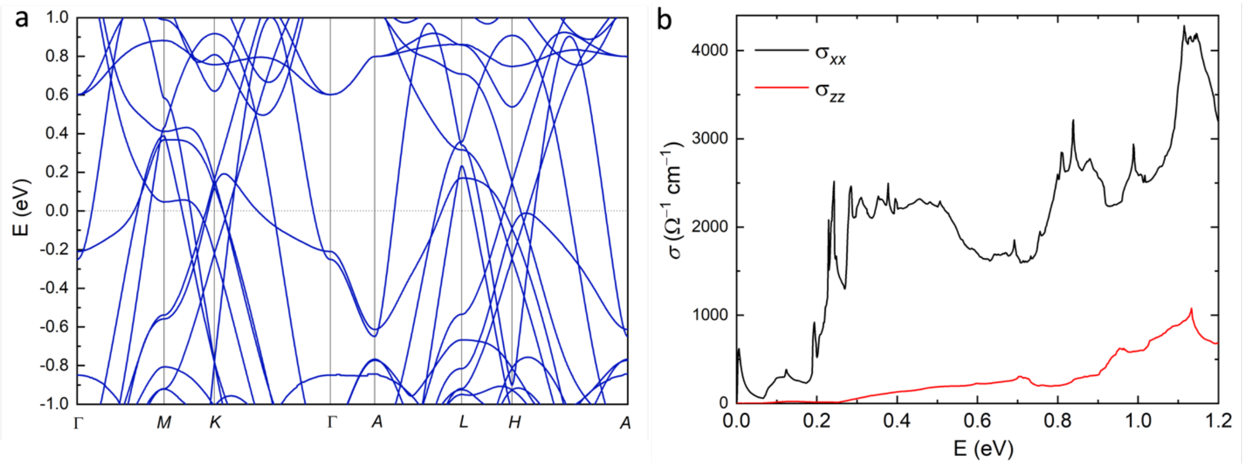

Supplementary Figure 12 | (a) DFT+U calculated band structure of CsV_3Sb_5, with the Fermi level set at 0 eV. (b) DFT+U calculated real parts of the in-plane (σ_{xx}) and out-of-plane (σ_{zz}) optical conductivity.

Standard DFT methods rarely treat d-orbitals accurately. We thus employed the DFT+U approach to treat the on-site Coulomb interactions on the localized d-states of vanadium atoms (SFig. 12). Since the exact value of the on-site Coulomb repulsion U is not known *a priori*, we employed U = +2 and +4 eV to cover the typical range of values generally accepted for vanadium [S11-S13]. The on-site Hund's coupling parameter J was set to 0.4 eV, and the double-counting correction in the atomic limit was used. Introducing the U term in DFT calculations has already improved the agreement between the dielectric function and our experimental results. As shown in SFig. 13, the calculated dielectric function exhibits a positive sign in the experimental energy window. However, to make a more direct comparison with our experimental data, we need to account for the intra-band transitions that are present in the experimental ε_1 values but missing from the DFT+U calculations, which considered the inter-band transitions only.

To address this issue, we have simulated the c-axis intra-band response and added it to the dielectric function obtained from the DFT+U calculation. Briefly, we used the following formula to simulate the real part of the dielectric function for intra-band transitions assuming a single Drude contribution:

$$\varepsilon_1(\omega) = 1 - \frac{\omega_p^2}{\omega^2 + \gamma^2} \quad (10)$$

The out-of-plane plasma frequency, ω_p = 0.62 eV, is obtained from the DFT+U calculations. Together with the experimental d.c. conductivity (σ_{dc}) [S14] is used to determine the damping rate (γ) of the carriers. The resulting dielectric function is shown in the main text Fig. 5b and SFig. 13, providing a more direct comparison with our experimental data.

Finally, we discuss the obtained σ_{xx} using both DFT+U and DFT methods. As illustrated in SFig. 11 & 12, there are noticeable differences in the σ_{xx} obtained from the DFT+U calculation when compared to the σ_{xx} obtained from the DFT calculation. In short, these disparities arise due to the high sensitivity of low-energy inter-band transitions to the inclusion of the on-site U term [S15]. Notably, when U = 4 eV, the corresponding σ_{xx} undergoes a dramatic change, particularly in the

low-energy regime between 0 eV and 0.2 eV, leading to a description that may not be as realistic when compared with the far-field FTIR generated in-plane spectra [S7]. This discrepancy can be quantitatively explained by considering that the DFT+U calculated σ_{xx} only contains the inter-band contributions. The inclusion of the Drude part intra-band contributions is expected to enhance the low-energy spectra, thereby improving the DFT+U based σ_{xx}. However, it is still likely that the DFT+U generated σ_{xx} will not perfectly match the experimental results, unlike the DFT generated σ_{xx}. This discrepancy suggests that the electronic correlations are more pronounced along the out-of-plane direction, while the in-plane direction predominantly retains its non-correlated characteristics. As a result, the standard DFT method more effectively captures the behavior of the optical spectra σ_{xx} in the in-plane direction [S7].

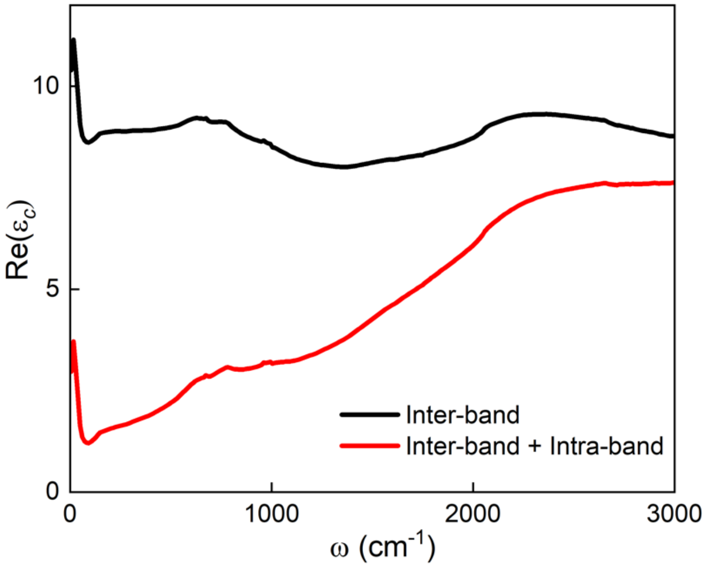

Supplementary Figure 13 | DFT calculated optical spectra of Re(ε_c) at U = 4 eV by considering both inter-band and intra-band contributions.

Supplementary References

S1. Shao, Y. *et al.* Infrared plasmons propagate through a hyperbolic nodal metal. *Science Adv.* **8**, eadd6169 (2022).

S2. Ni, G. X. *et al.* Ultrafast optical switching of infrared plasmon polaritons in high-mobility graphene. *Nature Photon.* **10**, 244–247 (2016).

S3. Dai, S. *et al.* Tunable phonon polaritons in atomically thin van der Waals crystals of boron nitride. *Science* **343**, 1125-1129 (2014).

S4. Fei, Z. *et al.* Gate-tuning of graphene plasmons revealed by infrared nano-imaging. *Nature* **487,** 82-85 (2012).

S5. Ni, G. X. *et al*. Fundamental limits to graphene plasmonics. *Nature* **557**, 530-533 (2018).

S6. Sunku, S. S. *et al.* Photonic crystals for nano-light in moiré graphene superlattices. *Science* **362**, 1153-1156 (2018).

S7. Uykur, E. *et al.* Low-energy optical properties of the nonmagnetic Kagome metal CsV_3Sb_5. *Phys. Rev. B* **104**, 045130 (2021).

S8. Blaha, P. *et al.* WIEN2k: An APW+lo program for calculating the properties of solids. J. Chem. Phys. 152, 074101 (2020).

S9. Perdew, J. P., Burke, K., Ernzerhof, M. Generalized Gradient Approximation Made Simple, Phys. Rev. Lett. 77, 3865 (1996).

S10. Ambrosch-Draxl, C. and Sofo, J. Linear optical properties of solids within the full-potential linearized augmented planewave method. Comput, Phys. Commun. 175, 1 (2006).

S11. Lutfalla, S., Shapovalov, V. & Bell, A. T. Calibration of the DFT/GGA+ U method for determination of reduction energies for transition and rare earth metal oxides of Ti, V, Mo, and Ce. J. Chem. Theory Comput. 7, 2218–2223 (2011).

S12. Weber, C. et al. Vanadium dioxide: A Peierls-Mott insulator stable against disorder. Phys. Rev. Lett. 108, 256402 (2012).

S13. Calderon, C. E. et al. The AFLOW standard for highthroughput materials science calculations. Comput. Mater. Sci. 108, 233–238 (2015).

S14. Xiang, Y. *et al.* Nature Commun. 12, 6727 (2021).

S15. Uykur, E., Ortiz, B. R., Wilson, S. D., Dressel, M. & Tsirlin, A. A. Optical detection of the density-wave instability in the Kagome metal KV_3Sb_5. npj Quan. Mater. 7, 16 (2022).

S16. Z. Sun, M. M. Fogler, D. N. Basov, and A. J. Millis, Collective modes and terahertz near-field response of superconductors. Phys. Rev. Research 2, 023413 (2020).
